# A Survey of Screen Media Access and Use in Primary School Children’s Households

**DOI:** 10.3390/children10010028

**Published:** 2022-12-23

**Authors:** Ashley E. Hinten, Kristina Wolsey, Annette M. E. Henderson, Damian Scarf

**Affiliations:** 1 Department of Psychology, University of Otago, Dunedin 9054, New Zealand; 2 School of Psychology, University of Auckland, Auckland 1010, New Zealand

**Keywords:** technology use, screen media, household life, primary school students, Aotearoa New Zealand

## Abstract

Our primary aim was to gain a better understanding of current technology availability and use in the homes of primary school children. The online-accessible questionnaire was made available for families with a child enrolled at primary school, with over 300 families participating. The results suggest that it is common for children to be introduced to screen media early in life and that they watch a wide range of content. While many families have rules regarding their children’s technology use, screen media is a significant part of their lives, with many children exceeding the recommended two hours of viewing per day. Future research could investigate whether media access and use differ between ethnic and socio-demographic groups, and whether changes have occurred as a result of the coronavirus pandemic.

## 1. Introduction

In Aotearoa New Zealand, the traditional television set (TV) started to appear in homes around 1960 [1]. TVs were, however, extremely expensive (i.e., 10 times the average weekly wage). Usually, one household on each street owned a TV, and neighbors went to their house to watch it. Dave Howell, a TV technician in Dunedin when broadcasts there first began, said “The novelty of it all was fascinating and we had people who stood outside the shops in the pouring rain, under the verandas, watching for the whole four hours of transmission each night” [1]. In quick time, TVs became more common in homes, creating the first generation of children in Aotearoa New Zealand, who experienced TV as an impromptu babysitter [2,3,4,5,6] or “digital pacifier” [7].

Fast forward several decades, and the TV is no longer the dominant form of screen media. Indeed, the last 70 years have seen a proliferation in the number and type of screen media devices available (e.g., desktop/laptop computers, smart phones, tablets, etc.) and the introduction of the internet (e.g., broadband/fiber internet connections, mobile data, etc.) [8]. Simultaneously, there has been a striking increase in child-directed media, both with regard to content (e.g., apps, games, TV shows and entire channels, VCR tapes/DVDs, etc.) and devices (e.g., computer keyboards and mice, Leapfrog educational toys, etc.) [7,9,10,11]. This proliferation of screen media means that children are now exposed to more types of media at an earlier age. For instance, it has been estimated that while children started watching TV when they were approximately 4 years old in the 1970s, children now begin closer to 4 months old [12].

With devices gaining popularity and becoming more mainstream, the American Academy of Pediatrics (AAP) [13] held meetings to develop recommendations for parents, teachers, and doctors around media exposure and use. Similar to their stance on TV viewing, they endorsed their earlier recommendation that children should not watch any screen media before their second birthday [14], and added a recommendation of a maximum of 1 h/day for 2- to 5-year-olds. Closer to home, the Australian government advised that children aged 2 to 5 years old only watch screen media for up to 1 h/day [15], and that 5- to 12-year-olds limit their screen media time to 2 h/day [16]. Similarly, although they provide no recommendations for children younger than 5 years old, the Aotearoa New Zealand Ministry of Health guidelines suggest 5- to 17-year-olds use screen media devices recreationally for a maximum of 2 h/day [17].

Not surprisingly, parents’ awareness and application of these types of recommendations vary widely [13,18]. For example, results from Common Sense Media surveys in the US demonstrated that only 20% of parents reported they knew of the AAP recommendations [8]. Further, children under 24 months old were exposed to screen media, spending an average of 29 min watching TV, 7 min watching DVDs, and 7 min on mobile devices per day (total of 42 min/day).

While there is a wealth survey of data from the US on media use in children, there is a need to carry out country-specific research, as (1) viewing habits may differ as a function of culture and (2) research suggests the relationship between screen media use and other variables may differ in country-specific ways [19,20]. For example, Christakis et al. [21] reported that for children in the US, more hours spent watching TV daily, at both 1 and 3 years old, was positively associated with attention problems at 7 years old. In contrast, Obel et al. [20] failed to replicate these findings with a sample of children using data from the Danish Aarhus Birth Cohort. Specifically, they reported no relationship between viewing time at 3.5 years old and attention or behavioral problems at 11 or 12 years old. Mixed findings have also been reported with data sets from Japan [22] and Aotearoa, New Zealand [23].

Moreover, public recommendations for media use may influence children’s viewing behaviors. Parents in countries with public recommendations (e.g., the US or Aotearoa New Zealand) may dissuade or limit their child’s use of screen media, while parents in countries without public recommendations (e.g., the UK or Mexico) might have more positive perspectives on screen media and not restrict their child’s access [24,25]. Supporting this idea, children from Mexico in the Hurwitz et al. [24] study used screen media devices over 2 h more than children from the US on weekdays and weekend days. Similarly, Mullan and Hofferth [25] reported higher screen media rates for children in the UK than in the US; however, the time difference between samples was smaller (i.e., 20–30 min).

There are relatively few sources of data on the media viewing habits of children in Aotearoa New Zealand. One exception to this is the Dunedin Multidisciplinary Health and Development Study (DMHDS). The DMHDS has followed 1037 people from Dunedin since their births between 1972 and 1973. Based on DMHDS data, in the late 1970s and early 1980s, children in Aotearoa New Zealand watched an average of 2.05 h of TV per day [26,27]. A more informative picture of viewing habits in the current media environment can be drawn from the Growing Up in New Zealand Study (GUiNZS). GUiNZS is a cohort study of children born between 2009 and 2010 in Auckland and the Waikato [28]. Based on the GUiNZS data, at 2 years old, the majority of children have watched screen media, with approximately 1 in 3 viewers watching more than 2 h/weekday [23].

As far as we are aware, the DMHDS and GUiNZS are the only studies that have assessed children’s screen media exposure in Aotearoa New Zealand. Moreover, given the rapidly changing media landscape, it is important to provide timely data on children’s media habits. To this end, the aim of the current study was to assess screen media access and viewing behaviors of primary school-aged children. Parents were invited to take part in the study when visiting the Adolescent Behaviour and Child Development (ABCD) Lab at the University of Otago or the Early Learning Lab (ELLA) at the University of Auckland. In addition, a smaller number of parents were recruited through a Southland primary school and Facebook advertisements. We were particularly interested in the technology available in the children’s homes, the families’ rules around screen media access and use, the children’s favorite shows, and how much time children delegated to screen media. In addition, given previous research linking media use and attention [21], we included the Child Behavior Questionnaire for 3- to 7-year-olds (CBQ) [29,30] and the 25-item Strengths and Difficulties Questionnaire for 4- to 17-year-olds (SDQ) [31]. Given the inconsistent results noted above, we made no specific predictions about the relationship between media use and CBQ and SDQ scores.

## 2. Materials and Methods

Parents were provided with information about the study, and, after agreeing to participate, they were asked to provide their consent either on paper or electronically. Parents were then presented with the questionnaire and were asked to respond to the questions with one of their children in mind. Responses were received between July 2018 and January 2020. The current project was reviewed and approved by the University of Otago Human Ethics Committee.

### 2.1. Questionnaire

#### 2.1.1. Demographics

To ensure the questionnaire was not completed for the same child multiple times, parents/guardians were asked to provide the child’s full name (data were subsequently de-identified, with a unique ID added for each child). Parents were then asked to provide their child’s date of birth, ethnicity, gender, and year at primary school (i.e., Year 1 through 6). Using a 6-point scale (i.e., 1 Did not attend high school, 2 Did some high school, 3 Completed high school, 4 Did some tertiary education, 5 Completed a diploma or undergraduate degree [e.g., BSc/BA], and 6 Completed a postgraduate degree [e.g., Master’s or Doctorate]), participants were asked to report the highest level of education achieved by each parent.

#### 2.1.2. Technology Use

Several questions were asked about the technology available in their homes and family rules around screen media (e.g., what devices and sites/applications they are permitted to watch on). Parents were also asked to report their child’s favorite show(s) and the age at which their child began watching TV. In addition, parents were asked to complete two 24 h media diaries (i.e., one for a weekday and one for a weekend day of their choice). The parents were asked to record what video media their child watched (e.g., standard TV, DVDs, streaming on Netflix and YouTube). Time-use diaries are popular tools for media research, and there are many examples in recent literature [24,25]. Our time-use diaries were developed following personal communication with Professor Rachel Barr [19]. She has been involved in the development of the Comprehensive Assessment of Family Media Exposure (CAFE) Consortium’s time-use diary tool [32].

The diary entries were parsed into broad activity categories: sleeping/resting, eating/drinking, bathroom/grooming, digital media use, reading, homework, non-active play (e.g., toys or puzzles), active play (e.g., outings at the park), unclear play (e.g., play date at friend’s house), childcare/school, extracurricular activities, church, travelling/errands, and chores at home. Responses where parents wrote they did not know or left slots blank were also coded.

Minimal changes were made to the parents’ reports. If they entered two or more activities in the same 30 min slot, the coder discerned the main activity and coded it as that. For example, if at 8:30 a.m. a parent reported their child was “playing during the drive to school”, it was coded as travelling because transport was the main activity. If two or more activities in one slot happened sequentially (e.g., at 2:30 p.m. a parent reported they “went home, then nap time” for one slot), the slot was equally split between the activities (i.e., half to travelling and half to resting in this example). If media use occurred alongside one or more other activities, and one of the activities was more passive (e.g., snacking while watching TV), the media was prioritized, as the viewing was the main activity and would likely have the child’s focus. If the slot reported something along the lines of “watching TV and playing,” however, the slot was split equally among the activities (i.e., half to screen media and half to non-active play in this example) because the main activity was not obvious.

Multiple difficulties arose when trying to code the media diaries. The instructions asked parents to include what their child was doing for each 30 min slot for each day, regardless of whether they were watching screen media or not. Multiple parents (a) only noted when their child was watching screen media; (b) missed slots on the diaries, so it was unclear what the child was doing; (c) did not provide the requested details when their child did view media (e.g., no mention of what they watched, what device they were watching on, and/or who they were watching with); and/or (d) indicated they thought the day they were reporting on was atypical, even though their description indicated otherwise (e.g., a parent says the day was not typical because of the weather, but this would generally be considered a normal day as it was a typical rainy day).

In these cases, the parents were contacted to request the missing details. If the parent only included media in their time-use diaries and they did not amend the diaries, their time-use data were excluded from the analyses. Otherwise, diaries were retained if most of the requested information was provided (e.g., an activity was provided for each slot, but they left out the device their child was using to watch media, or they had a small number of blank slots). Coder-judged typicality variables were also added for when the coder disagreed with the parent’s description of whether the day was typical or not. Participants were removed from the analysis due to (a) only reporting media engagement (weekday *n* = 16, weekend day *n* = 15), (b) leaving multiple 30 min sections blank in the diary (weekday *n* = 7; weekend day *n* = 17), and/or (c) not reporting an appropriate date for the diary, such as providing a weekday date for a weekend day diary (weekday *n* = 10, weekend day *n* = 13). Thus, in total, 68 weekday and 82 weekend day diaries were removed, leaving 288 weekday diaries and 274 weekend day diaries.

Of the weekday diaries, 228 of the diary days were judged to be typical days for each child, 48 were atypical, and typicality was unclear for 12 (i.e., the parent did not circle an option, or the coder was unsure how to code it after reading the parent’s explanation). Further, 276 were completed for a school term date, and 12 were completed for a school holiday date. Of the weekend day diaries, 204 of the diary days were judged to be typical days for each child, 62 were atypical, and typicality was unclear for 8. Further, 218 were completed for a school term date, and 38 were completed for a school holiday date, 5 completion dates were left blank, and 8 were unclear (i.e., schools have varying term start dates; thus, it was difficult to discern if the date was during term without knowing each child’s school).

#### 2.1.3. Behavior Questionnaires

Parents completed the very short 36-item version of the CBQ [29,30] and the 25-item SDQ [31]. The SDQ’s Hyperactivity subscale and the CBQ’s Effortful Control subscale include items measuring executive function skills (e.g., attention, goal persistence, impulsivity, inhibitory control, and planning); hence, these were used as measures of cognitive development in the analysis.

The CBQ items are equally split across three subscales: Surgency, Negative Affect, and Effortful Control. An example Effortful Control item is “Prepares for trips and outings by planning things s/he will need.” Parents were asked to rate how well each item describes their child on a 7-point scale from 1 Extremely Untrue to 7 Extremely True, or parents could answer Not Applicable. The ratings for each subscale were averaged by the number of subscale items they answered to give their average rating (range 1 to 7). Both Putnam and Rothbart [29] (Surgency *α* = 0.78, Negative Affect *α* = 0.74, Effortful Control *α* = 0.69) and Sleddens et al. [33] (Surgency *α* = 0.75, Negative Affect *α* = 0.72, Effortful Control *α* = 0.74) report good internal consistency for the very short form of the CBQ.

The SDQ items are equally split across five subscales: Emotional, Conduct, Hyperactivity, and Peer problems subscales, and a Prosocial subscale. An example Hyperactivity item is “Constantly fidgeting or squirming.” Parents answered whether each item accurately described their child on a 3-point scale from 0 Not True to 2 Certainly True (range 0 to 10 per subscale). The problems subscales are combined to give a Total Difficulties score (range 0 to 40). Stone, Otten, Engels, Vermulst, and Janssens’s [34] review of international studies suggests good internal consistency for parental completions for 4- to 12-year-olds.

### 2.2. Analyses

Data on child and parent demographics, technology access, and the children’s time-use were similarly summarized using means, Standard Deviations (*SD*s), ranges, frequency counts, and percentages. Data on household rules and top-watched shows were summarized using frequency counts and percentages. Two-tailed Pearson correlations were used to explore the relationships between child and parent demographics, child executive function, and children’s time allocation between a subset of reported activities (i.e., screen media, reading, homework, play, and extra-curricular activities) on weekdays and weekend days. Time-use diaries were coded in Excel, and SPSS was used for all analyses.

## 3. Results

### 3.1. Demographics

The questionnaire was completed for 181 male and 175 female children (50.84% male). Children had a mean age of 6.5 years (*SD* = 0.94, range: 4.27 to 10.84). The majority of participants were recruited through the ABCD and ELLA labs (*n* = 222), followed by Facebook (*n* = 116) and the primary school (*n* = 18). Using priority coding, most participants were of European descent (i.e., Pākehā; *n* = 244), followed by Māori (*n* = 50), Asian (*n* = 29), Pacific Islander (*n* = 5), African (*n* = 3), and Other (*n* = 3; two identifying as Latino and Japanese, and one as Pākehā, Māori, and Dutch). Further, 22 participants provided a non-specific answer to the ethnicity question, such as ‘Kiwi’ or ‘New Zealander’. These ambiguous answers were coded as ‘Other’.

The vast majority of the children (341; 95.79%) were within the CBQ’s 3 to 7 years old age bracket, 13 children (3.65%) were 8 years old or older, and ages were unknown for 2 participants (0.56%; implausible birth years were provided). Cronbach alpha values for the CBQ subscales were acceptable for the whole sample (Surgency *n* = 348, *α* = 0.77; Negative Affect *n* = 339, *α* = 0.78; Effortful Control *n* = 332, *α* = 0.69), as well as the 3 to 7 years old (Surgency *n* = 335, *α* = 0.77; Negative Affect *n* = 326, *α* = 0.78; Effortful Control *n* = 319, *α* = 0.70) and 8 years old and older subsets (*n* = 13; Surgency *α* = 0.79; Negative Affect *α* = 0.62; Effortful Control *α* = 0.62).

In terms of the mothers’ educational achievement, 1 (0.28%) did not attend high school, 30 (8.42%) did some high school but did not graduate, 24 (6.74%) graduated from high school, 63 (17.70%) started tertiary education but did not complete their degree, 182 (51.12%) graduated university with a diploma or undergraduate degree (e.g., Bachelor of Science/Art), and 56 (15.73%) graduated university with a postgraduate degree (e.g., Master’s or Doctorate). For fathers, 4 (1.12%) did not attend high school, 58 (16.29%) did some high school but did not graduate, 46 (12.92%) graduated from high school, 92 (25.84%) started tertiary education but did not complete their degree, 122 (34.27%) graduated university with a diploma or undergraduate degree, and 34 (9.55%) graduated university with a postgraduate degree.

### 3.2. Technology Access

The average age for starting to watch media was 22 months (*SD* = 12, range = 0 to 48). Only parents of 11 children (3.08%) reported an onset age at 4 months old or younger. Reflecting the changing patterns of media use across time, only 17 (4.78%) parents reported that their child had never watched TV. Five of these participants did, however, provide data on their child’s top-watched shows, suggesting their child watched media exclusively using other screen media devices. With respect to viewing time, most children were allowed to watch in the late afternoon from 3:00 p.m. to 6:00 p.m. (280; 78.65%), followed by early morning before 10:00 a.m. (185; 51.97%), then evening from 6:00 p.m. (97; 27.25%), early afternoon from noon to 3:00 p.m. (23; 6.46%), and then late morning (20; 5.62%). The low responses for these latter two categories were not surprising, as children in Aotearoa New Zealand are typically at school during these times.

Many of the families had multiple technological devices available in their home. Specifically, 346 (97.19%) had a TV, 333 (93.54%) had a smart phone, 289 (81.18%) had a tablet, 277 (77.81%) had a laptop computer, 185 (51.97%) had a gaming console (e.g., PlayStation, Xbox, etc.), and 107 (30.06%) had a desktop computer. In regard to the number of these devices families had at home, 4 (1.1%) had only one device, 18 (5.1%) had two different devices, 55 (15.4%) had three, 103 (28.9%) had four, 136 (38.2%) had five, and 40 (11.2%) had all six listed. Five (1.40%) participants also indicated another type of device (e.g., Leapfrog talking book or virtual reality headset). In terms of whether the children were allowed to watch media on the devices in their homes, 315 (88.48%) were allowed to watch media on a TV, 145 (40.73%) on the tablet, 70 (19.66%) on the smart phone, 40 (11.24%) on the laptop computer, 36 (10.11%) on the gaming console, and 20 (5.62%) on the desktop computer, while 6 (1.69%) were not allowed to watch on any device.

Streaming sites and applications were also popular: 270 (75.84%) had Netflix, 233 (65.45%) had YouTube, 47 (13.20%) had Lightbox, 20 (5.62%) had TVNZ+, 13 (3.65%) had Sky or Sky Go, 8 (2.25%) had Neon, 5 (1.40%) had Hei Hei, 5 (1.40%) had Amazon Prime, 5 (1.40%) had downloaded content, and 1 each (0.28%) had Animelab, Disney+, Kodi box, Baobao bus, Reading Eggs (an online course to teach children to read), or Plex. Disney+ was released in Aotearoa New Zealand at the end of data collection in November 2019, which helps explain the low number of its users in this participant sample. Its prevalence has likely increased in the years since its release. Since the end of this study, Hei Hei was added as a show category to TVNZ+ in May 2020. Hence, more children may regularly access Hei Hei content now. While Reading Eggs was only mentioned in one participant’s questionnaire, multiple participants included it in their time-use diaries. This reflects that some parents did not consider it or other educational games when answering this questionnaire item.

In a similar vein, children rarely watched DVD or Blu-ray discs, with 117 (32.87%) never having watched them and 166 (46.63%) only having watched a DVD a couple of times a month. A smaller subset of 57 (16.01%) participants watched a couple of times a fortnight, while only 16 (4.49%) watched them multiple times a week.

### 3.3. Household Viewing Rules

For the 343 families whose children were allowed to watch media, most (309; 90.09%) had at least one household viewing rule. Five families indicated they had family rules, but chose not to elaborate on these. The most common rule was related to the times of day children were allowed to watch (164; 53.07%). Many children were not allowed to watch in the morning before school, while others had narrow windows during which they were allowed to watch (e.g., while their parents cooked dinner). The second most popular rule was conditional viewing (122; 39.48%), whereby children were not allowed to watch unless a set of parameters were met: for example, having to change out of their school uniform and tidy away their school bag first, having to complete their homework and/or chores first, and only viewing if the weather was poor and they could not play outside.

The third most popular rule regarded how long children were allowed to watch for, with many parents (114; 36.89%) enforcing a set time per viewing event (e.g., one episode at a time) or per day (e.g., only two episodes or one movie per day). Less common rules were to do with the day of the week (71; 22.98%) and the type of content (53; 17.15%).

### 3.4. Top-Watched Shows

Across the sample, parents reported 977 different top-watched show titles. We deduced the top-watched shows using the three top-watched shows parents reported (see Table 1). Comparing these to the Nielsen list of American children’s favorite shows (see Taggart, Eisen, & Lillard [35]), six of the shows overlapped (i.e., Paw Patrol, PJ Masks, Lego Ninjago, Teen Titans Go!, My Little Pony, and Peppa Pig). Perhaps reflecting the difficulty of tracking viewing on non-traditional formats, with respect to YouTube, many parents simply reported ‘YouTube’ (19; 38.78%). Reflecting the greater diversity of content on these formats, for those that did provide information on content, there were eight reports of hobby-related videos, such as arts and crafts, science experiments, and music. Children also watched gaming videos, video blogs (i.e., vlogs), and toy unboxings.

### 3.5. 24-Hour Time-Use Diaries

Media use was the fifth most popular activity; participants watched an average of 0.98 h per weekday (*SD* = 0.89, range: 0 to 4). Additionally, 120 (41.67%) of the correlational questionnaire participants watched more than 2 h on the reported weekday. On weekends, time delegation to screen media use was second only to sleeping. Participants spent an average of 2.63 h watching media per weekend day (*SD* = 1.69, range: 0 to 7.42). On average, children watched almost three times as much screen media on weekend days than weekdays. Moreover, 225 (82.12%) watched more than 2 h on the reported weekend day; this suggests screen viewing is higher during the weekend than the school week. Table 2 includes the descriptive statistics for how many hours the children spent on each activity on average on a weekday versus a weekend day.

### 3.6. Correlations

In light of the sheer number of correlations possible with this data set, relations were restricted to the main measures of interest and are reported in Table 3. The active, non-active, and unclear play codes were collapsed into a single play code.

#### 3.6.1. Demographics

Maternal education and paternal education were positively related to one another (*r* = 0.525, *p* = 0.000), as well as to higher scores on the CBQ’s Effortful Control subscale (maternal *r* = 0.150, *p* < 0.001; paternal *r* = 0.239, *p* < 0.001). Further, paternal education was negatively correlated with Hyperactivity scores on the SDQ (*r* = −0.154, *p* < 0.001). Maternal education was also related to more reading (weekday *r* = 0.276, *p* < 0.001; weekend *r* = 0.197, *p* < 0.001), more play (weekday *r* = 0.123, *p* = 0.036; weekend *r* = 0.130, *p* = 0.032), and less screen media on weekdays (*r* = −0.170, *p* = 0.025). Similarly, higher paternal education was associated with more weekday and weekend reading (weekday *r* = 0.231, *p* < 0.001; weekend *r* = 0.190, *p* < 0.001), more weekday playing (*r* = 0.119, *p* = 0.044), and less screen media during the school week (*r* = −0.233, *p* < 0.001). Unlike maternal education, paternal education was also positively related to weekend extracurricular activities (*r* = 0.119, *p* = 0.049).

#### 3.6.2. Executive Function

Scores on the two executive function questionnaire subscales were significantly intercorrelated, with higher CBQ Effortful Control scores (i.e., higher EF) negatively related to higher SDQ Hyperactivity scores (i.e., lower EF; *r* = −0.395, *p* < 0.001). Higher EF performance on these measures (i.e., higher Effortful Control score and lower Hyperactivity score) were also negatively correlated with weekend screen media viewing (Effortful Control *r* = −0.171, *p* = 0.026; Hyperactivity *r* = 0.158, *p* = 0.040). Higher Effortful Control was also correlated with more reading (weekday *r* = 0.135, *p* = 0.022; weekend *r* = 0.146, *p* = 0.015).

#### 3.6.3. Weekday Diary

Watching more screen media on weekdays was positively correlated with watching it during the weekend (*r* = 0.489, *p* < 0.001). Weekday screen media was also negatively related to reading (weekday *r* = −0.176, *p* = 0.021; weekend *r* = −0.204, *p* = 0.009) and weekday extracurricular activities (*r* = −0.164, *p* = 0.032). Similarly, reading during the week was strongly and positively correlated with weekend reading (*r* = 0.701, *p* < 0.001). While it was positively associated with more weekday play (*r* = 0.163, *p* = 0.005), it was negatively related to weekday homework (*r* = −0.162, *p* = 0.006) and weekend screen media viewing (*r* = −0.276, *p* < 0.001).

Doing homework during the school week was negatively associated with time spent playing (weekday *r* = −0.172, *p* = 0.003; weekend *r* = −0.191, *p* = 0.002) and reading (weekday *r* = −0.162, *p* = 0.006; weekend *r* = −0.131, *p* = 0.034). Playing during the week was positively correlated with weekend play (*r* = 0.211, *p* = 0.001) and weekend reading (*r* = 0.245, *p* < 0.001), but negatively correlated with extracurricular activities (weekday *r* = −0.323, *p* < 0.001; weekend *r* = −0.144, *p* = 0.020) and weekend screen media (*r* = −0.191, *p* = 0.015). Time spent on extracurricular activities during the week was also positively associated with doing similar activities during the weekend (*r* = 0.271, *p* < 0.001).

#### 3.6.4. Weekend Day Diary

In addition to the weekend day relationships outlined above, weekend screen media viewing was negatively correlated with reading (*r* = −0.327, *p* < 0.001), playing (*r* = −0.239, *p* < 0.001), and extracurricular activities (*r* = −0.169, *p* = 0.028).

#### 3.6.5. Household Viewing Rules

Out of interest, we also looked at whether the rule types were related to children’s time-use. Having rules regarding what day children could watch led to less weekday (*r* = −0.294, *p* < 0.01) and weekend day viewing (*r* = −0.169, *p* < 0.05), while the conditional rule led to more weekday (*r* = 0.233, *p* < 0.01) and weekend day viewing (*r* = 0.194, *p* < 0.05). Further, the day rule was related to more weekday active play (*r* = 0.121, *p* < 0.05) and more weekend reading (*r* = 0.192, *p* < 0.01), while the viewing length rule was related to more weekday active play (*r* = 0.162, *p* < 0.01), and the time rule was related to more weekend extracurricular activities (*r* = 0.168, *p* < 0.01).

## 4. Discussion

The primary aim of the current study was to attain more recent data on screen media access and viewing behaviors of primary school-aged children. Although we had no set hypotheses, the questionnaire responses are consistent with earlier research conducted in Aotearoa New Zealand and abroad. That is, media use is a significant part of the home lives of many children, with >98% of children allowed to watch media of one form or another. This mirrors Himmelweit, Oppenheim, and Vince’s [36] and Schramm, Lyle, and Parker’s [37] early difficulty in finding participants who had never watched TV in the US.

It was common for children to be introduced to screen media just before their first birthday. The earliest reported age was birth, with these parents reporting they could not remember a time when their child did not watch screen media. The oldest reported age was 48 months old. This aligns with US research, where it is common for parents to familiarize their children with screen media devices before their first birthday, possibly to keep children occupied while parents complete errands or, more recently, to calm children down in medical settings [7]. Our findings, however, are not consistent with Radesky and Christakis’s [12] claim that children growing up in the 2010s begin watching screen media when they are 4 months old, compared to 4 years old in the 1970s. Indeed, only parents of 11 children (3.08%) reported an onset age at 4 months old or younger.

On average, children spent 0.98 h (*SD* = 0.89) watching screen media on weekdays. Children watched almost three times as much screen media on weekends, with an average of 2.63 h (*SD* = 1.67). Of course, our reported viewing times are averages, thus the daily viewing times are more diversified across participants. Consequently, we also looked at how many children exceeded the recommended two hours’ screen media viewing per day cf. [14]. We were especially interested in this, as past research suggests parents are not aware of these guidelines [8,38]. On weekdays, 4 in 10 participants (41.67%) exceeded this recommendation, but on weekends, 8 in 10 exceeded it (82.12%).

The participants’ screen media use was similar to samples of children from the US in recent time-use research. For example, 8- to 17-year-olds in the Mullan and Hofferth [25] study used screen media for an average of 1.48 h on weekdays and 2.44 h on weekend days. Hurwitz et al. [24] participants were of a closer age to the participants in the current study (i.e., 2.5 to 8 years old); however, they watched more on weekdays (1.71 h) and less on weekend days (2.31 h). We cannot conclude that screen media rates for children in Aotearoa New Zealand are low because there are public recommendations. We did not ask parents whether they were aware of the Ministry of Health’s guidelines, or how they decided on their family rules more generally (e.g., doctor’s recommendations). We expect that some children may be encouraged to join in extra-curricular activities (e.g., sport) instead of watching screen media because of the high value of sport in Aotearoa New Zealand.

Some of the trends from the correlational analysis reflect the Hurwitz et al. [24] US sample findings. For example, we also found a negative relationship between screen media use and play on weekends, as well as negative associations between weekday screen media and weekday reading, and weekend day screen media with both weekend reading and weekend play. Hurwitz et al. [24] also reported that screen media use was positively related to doing chores. This links to our finding that conditional rules (e.g., “My child can only watch TV after they finish their chores”) is related to higher viewing. In contrast, we did not replicate trends found in the Mexican sample (e.g., more screen media was not positively related to outdoor play). These comparisons suggest screen media culture in Aotearoa New Zealand may be more alike the US than the UK or Mexico.

While almost all of the participants had a TV set (97.19%), the types of devices have expanded, as evidenced in recent research [7,10]. The most common alternative screen media devices were smart phones (93.54%), tablets (81.18%), and laptop computers (77.81%). While TVs remain an at-home staple, many of the other popular devices are portable, allowing users to use them whenever and wherever they wish. Rideout (2017) reported increased uptake of smart phones (41% to 95%) and tablets (8% to 78%) from 2011 to 2017 as they entered the market to compete against TV, and our results show supporting evidence in Aotearoa New Zealand. Of course, having a range of devices at home (and apps on said devices) does not necessarily mean children can use them. Therefore, we also asked which of the devices in participants’ homes were open to their children for watching screen media content. Most children could watch TV (88.48%), followed by tablets (40.73%), smart phones (19.66%), laptop computers (11.24%), gaming consoles (10.11%), and then desktop computers (5.62%).

Interestingly, there was a vast number of titles reported for the top-watched shows questionnaire item. Indeed, 977 different titles were provided. Walters and Zwaga’s [39] sample’s top-watched titles were Bean: The Ultimate Disaster Movie, Jurassic Park, and The Simpsons, reported by 58%, 49%, and 46% of participants, respectively. In comparison, the sheer number of titles reported in the present study means that the top three shows were Paw Patrol, YouTube, and PJ Masks, reported by 7%, 5% and 3% of participants, respectively. This reflects the vast range of shows and movies that are child-directed or merely accessible to children (e.g., through streaming services). Further, Pokémon is the only title to appear on the top-watched list of Walter and Rwaga [39] and the present study. The percentage of children reporting it as their favorite show, however, has dropped from 38% to 1.9%. Outside of the top-watched lists, six titles overlap; specifically Jurassic Park, The Simpsons, Shortland Street, Pokémon, What Now? and Friends.

As portable devices, such as tablets, become more mainstream, it becomes increasingly difficult for parents to supervise their child’s media use. This may have contributed to the numerous incomplete diary entries. While the questionnaire did not explicitly ask whether parents had concerns about their child’s screen media use, the viewing rules can be used as a proxy measure, as they suggest parents are trying to limit or supervise their child’s media use. For example, 90.09% of the participating families had one or more household viewing rules. While time of day, conditional viewing, and length of exposure were popular restrictions, only 17.15% of participants had a rule about the type of content their child was allowed to watch (e.g., child- vs. adult-directed). In light of concern surrounding the length of time children view screen media [20,34] and the content children are watching [22,39,40], it is surprising that few parents had specific rules about how long their child was allowed to watch screen media or what content they were permitted to watch.

While there are many potential relationships to discuss, there are a few more interesting relationships we would like to highlight. For example, maternal and paternal education were intercorrelated, and both variables were separately, and significantly, positively correlated with having fewer screen media devices available at home, more family viewing rules, less weekday screen media viewing, as well as more reading and creative play on weekdays and weekend days. Additionally, paternal education alone was related to more weekend extracurricular activities. One potential explanation for this is that parents who are highly educated may encourage activities they themselves value and also perceive as both mentally stimulating and character developing (e.g., reading, creative play, and extracurricular activities, such as sports). Moreover, these parents likely have more resources to educate themselves on screen media effects. Indeed, if parental education is used as a crude measurement of socioeconomic status (SES; i.e., more education leads to a higher paying job), this result is similar to Rideout’s [8] finding that the amount of screen media viewed is negatively associated with annual family income.

There are three main limitations of this study. First, the low sample size makes it difficult to look at nuances between ethnic and socioeconomic groups. For example, children of ethnic minorities may be more likely to seek out animated shows about their ancestry (e.g., Taiohi Gods or Tales of Nai Nai, which focus on Māori and Asian mythology, respectively) or live action shows that include representation of the diverse ethnic groups of Aotearoa New Zealand (e.g., The Feijoa Club and Kea Kids News), resulting in different media exposure. Additionally, home access to devices and paid streaming platforms likely coincides with the families’ SES. For example, children from low SES families may have to share devices with other family members, leading to less viewing and/or more background or age-inappropriate TV exposure. Children from high SES families may have access to more streaming platforms, providing them with a wider range of professionally produced child-directed shows. Future research should prioritize recruiting a larger number of participants from a wide range of cultural groups to allow for more detailed analysis of their media exposure and use.

Second, the ethnic and socioeconomic distributions in the study sample do not reflect the wider population of Aotearoa New Zealand. The numbers of children who were identified as European, Māori, and Middles Eastern, Latin American, and African (MELAA) are similar to the numbers expected in the New Zealand Census [40]. However, the proportions of Pacific and Asian participants were disproportionately low, and the number of participants identifying as an ‘Other’ ethnicity was disproportionately high. These differences may be partially explained by our decision to priority code the participants’ ethnicities and assign them to one ethnic group. Moreover, children of parents who have pursued tertiary and/or postgraduate are over-represented in the study sample, while children of parents whose highest qualification was graduating high school or earlier are under-represented. Future research should also prioritize recruiting Pacific and Asian children, as well as children of parents who did not pursue higher education, to provide data on these groups.

Third, the coronavirus pandemic began shortly after these data were collected, meaning media use and access may have changed during lockdowns. For example, more pressure on parents who were working from home and also supervising their children’s schoolwork may have led to more reliance on media as a babysitter, leading to increased viewing. Similarly, instructions to stay at home and the temporary closing of places like public parks may have replaced time spent out of home with media viewing, especially due to media company responses to the pandemic (e.g., Storyline Online, where videos of celebrities reading children’s books were uploaded onto YouTube, and Disney+ releasing films online because movie theatres were closed). Further, financial strain due to parents being made redundant or having work hours reduced may have led some families to cancel streaming platform subscriptions, leading to reduced viewing. These data can act as a valuable baseline for current mid- and future post-pandemic investigations.

## 5. Conclusions

The current study provides insight into screen media’s place in homes in Aotearoa New Zealand. While most families had household rules to guide their children’s media exposure, many children exceeded recommendations of two hours viewing per day and weekdays and/or weekend days. Electronic time-use tools and passive sensing applications can complement parental reports and help overcome the limitations of manual coding, leading to highly detailed parental reports [32]. As screen media continues to evolve, it will be important to look further into the changing patterns of children’s media use, as well as the potential short- and long-term effects of media exposure on children’s development.

## Figures and Tables

**Table 1:** Top-watched television shows.

Rank	Show Title	Frequency	Percent
1	Paw Patrol *	70	7.2
2	YouTube (miscellaneous)	49	5.0
3	PJ Masks *	30	3.1
4	Lego (Miscellaneous) *	30	3.1
5	Teen Titans Go! *	28	2.9
6	My Little Pony *	25	2.6
7	Peppa Pig *	23	2.4
8	Barbie Life in the Dreamhouse	21	2.1
9	News	19	1.9
10	Pokémon	19	1.9

* Signifies the show is also popular in the US, as reported on the 2015 Nielsen top-rated list (see Taggart et al. [35]).

**Table 2:** Descriptive statistics in hours for time delegation to diary activities on weekdays versus weekend days.

	Weekday (*n* = 288)		Weekend Day (*n* = 274)	
Activity	Mean (SD ^1^)	Range	Mean (SD)	Range
Resting	11.36 (0.75)	8.50–13.50	11.42 (1.35)	0.05–15.50
School/Childcare	6.35 (1.08)	0.00–9.50	-	-
Eating	1.06 (0.50)	0.00–3.50	1.80 (0.84)	0.25–5.50
Traveling	1.00 (0.58)	0.00–4.00	1.78 (1.78)	0.00–10.00
Screen Media	0.98 (0.89)	0.00–4.00	2.63 (1.69)	0.00–7.42
Grooming	0.84 (0.38)	0.00–2.00	0.75 (0.51)	0.00–2.83
Non-Active Play	0.63 (0.70)	0.00–4.00	1.16 (1.56)	0.00–7.50
Reading	0.42 (0.51)	0.00–3.75	0.43 (0.61)	0.00–4.50
Extra-curricular	0.33 (0.54)	0.00–2.50	0.42 (0.78)	0.00–4.00
Active Play	0.32 (0.54)	0.00–2.50	1.31 (1.28)	0.00–5.50
Home Chores	0.24 (0.35)	0.00–2.50	0.46 (0.76)	0.00–6.25
Homework	0.18 (0.28)	0.00–2.00	0.02 (0.15)	0.00–1.50
Unclear Play	0.08 (0.31)	0.00–2.50	0.81 (1.67)	0.00–12.00
Church	-	-	0.08 (0.41)	0.00–3.00
Other	0.06 (0.21)	0.00–1.50	0.12 (0.46)	0.00–5.00
Blank	0.00 (0.03)	0.00–0.42	0.00 (0.04)	0.00–0.50

^1^ SD = Standard Deviation.

**Table 3:** Correlations between socio-demographic, behavior, and time-use variables.

Variables	1	2	3	4	5	6	7	8	9	10	11	12	13	14	15
1. Current age	-														
2. Maternal education	−0.028	-													
3. Paternal education	−0.125 *	0.525 **	-												
4. Age of TV introduction	0.130 *	0.039	0.001	-											
5. CBQ ^1^ Effortful Control	−0.048	0.150 **	0.239 **	−0.034	-										
6. SDQ ^2^ Hyperactivity	0.009	−0.091	−0.154 **	0.016	−0.395 **	-									
*Weekday*															
7. Screen Media	−0.096	−0.170 *	−0.233 **	−0.242 **	−0.134	0.058	-								
8. Reading	0.017	0.276 **	0.231 **	0.110	0.135 *	−0.115	−0.176 *	-							
9. Homework	0.025	−0.108	−0.110	−0.111	−0.064	0.073	0.025	−0.162 **	-						
10. Play	−0.150 *	0.123 *	0.119 *	0.075	0.069	0.020	−0.018	0.163 **	−0.172 **	-					
11. Extra-curricular	0.103	0.043	0.100	−0.054	−0.070	0.008	−0.164 *	−0.003	−0.090	−0.323 **	-				
*Weekend day*															
12. Screen Media	0.074	−0.141	−0.131	−0.336 **	−0.171 *	0.158 *	0.489 **	−0.276 **	0.062	−0.191 *	0.010	-			
13. Reading	0.081	0.197 **	0.190 **	0.111	0.146 *	−0.097	−0.204 **	0.701 **	−0.131 *	0.245 **	−0.029	−0.327 **	-		
14. Homework	0.039	0.100	0.046	−0.067	0.027	0.051	−0.142	−0.083	0.398 **	−0.095	0.000	0.022	−0.069	-	
15. Play	−0.043	0.130 *	0.099	0.059	0.006	0.011	−0.081	0.047	−0.191 **	0.211 **	0.034	−0.239 **	−0.011	−0.055	-
16. Extra-curricular	0.066	0.101	0.119 *	0.060	0.001	0.043	−0.098	−0.045	0.078	−0.144 *	0.271 **	−0.169 *	−0.029	0.003	−0.086

Two-tailed Pearson correlations; ^1^ CBQ = Child Behavior Questionnaire; ^2^ SDQ = Strengths and Difficulties Questionnaire; * *p* < 0.05, ** *p* < 0.01.

## Data Availability

The data presented in this study are available upon reasonable request from the corresponding author.
